# Procyanidin B2 mitigates behavioral impairment and protects myelin integrity in cuprizone-induced schizophrenia in mice†

**DOI:** 10.1039/c8ra03854f

**Published:** 2018-06-29

**Authors:** Hui Tian, Wanchun Sun, Qianying Zhang, Xiaofei Li, Ying Sang, Jian Li, Yunhui Niu, Hong Ding

**Affiliations:** Key Laboratory of Combinatorial Biosynthesis and Drug Discovery, Ministry of Education, Wuhan University School of Pharmaceutical Sciences, Wuhan University Wuhan Hubei P. R. China dinghong1106@whu.edu.cn +8613007162084

## Abstract

Numerous studies have suggested that neuropathological changes in schizophrenia may be related to damage to white matter or demyelination. Procyanidin B2, which is a constituent of many fruits such as grapes and strawberries, has various biological activities such as anti-inflammatory and anti-tumor activity, as has been reported. This study aimed to estimate the effects of procyanidin B2 on behavioral impairment and the protection of myelin integrity in a cuprizone-induced schizophrenia model. Mice were exposed to cuprizone (0.2% w/w in chow) for five weeks to induce schizophrenia-like behavioral changes and demyelination. Procyanidin B2 (20 or 100 mg kg^−1^ day^−1^) or vehicle was administered orally to mice after withdrawal from cuprizone. Behavioral impairment was detected with an open-field test, a rotarod test and a Morris water maze. Myelin integrity was assessed using LFB staining and MBP expression, including immunofluorescence and western blotting. In addition, enhancements in the expression of HO-1 and NQO1 suggested that procyanidin B2 may regulate oxidative homeostasis *via* promoting the translation of Nrf2 to the nucleus. Data indicated that procyanidin B2 could mitigate behavioral impairment and protect myelin integrity in the cuprizone-induced model *via* regulating oxidative stress by activating Nrf2 signaling.

## Abstract 2

Numerous studies have suggested that neuropathological changes in schizophrenia may be related to damage to white matter or demyelination.

## Graphical abstract

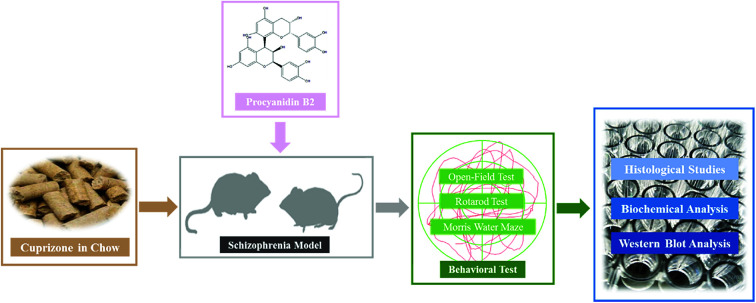

## 1. Introduction

Schizophrenia is a complex mental disorder with disturbances in emotion, perception, cognition and social function. Schizophrenia may affect approximately 1% of the population worldwide and is classified into positive symptoms, negative symptoms, and impairments in connectivity.^1–3^ Although the fundamental neuropathology of schizophrenia remains unexplained, an increasing number of studies have shown that dysfunctional neuronal communication may be a crucial basis of this disorder.^4^ Recently, numerous results indicated that cognitive disorder in schizophrenia is related to myelin sheath abnormalities and lesions in white matter.^1,5^ A myelin sheath, which consists of multiple coherent oligodendroglial cells, wraps an axon segment in the central nervous system (CNS).^6^ Imaging studies suggested that brains in schizophrenia patients exhibited degeneration of oligodendroglial cells and abnormal white matter, which indicated that abnormal white matter may be a potential cause of schizophrenia.^2,7,8^ Microarray analyses showed that genes were involved that are associated with the maturity of oligodendroglial cells and myelination.^9^ Therefore, an agent that protects myelin sheaths from demyelination could be a therapeutic target for schizophrenia.

Cuprizone (CPZ), which is a well-known copper-chelating agent, has been used to induce a mouse model of oligodendroglial loss and demyelination in the CNS owing to its toxic effects in the corpus callosum, hippocampus, and some white matter regions.^10^ Evidence indicated that CPZ would damage oligodendroglial cells but did not injure other types of cell.^11^ Mice with cuprizone intoxication exhibit schizophrenia-like behavior with behavioral abnormalities and neurological deficits.^12,13^ Quetiapine was reported to ameliorate schizophrenia-like behavior and protect myelin integrity *via* Notch signaling in a cuprizone-induced mouse model.^14^ In addition, studies suggested that the hydrogen peroxide concentration and activities of antioxidant enzymes were enhanced *via* the administration of cuprizone in the culture of oligodendroglial cells and a cuprizone-induced model.^15,16^ Increasing evidence proved that oxidative stress is an important component of the pathological mechanism of schizophrenia.^17–19^ Therefore, we speculated that oxidative stress may be a key cause of demyelination in schizophrenia.

Previous evidence indicated that oxidative stress may account for pathological changes in demyelination in a cuprizone-induced mouse model.^16,20^ Nuclear factor erythroid 2-related factor 2 (Nrf2) is a basic leucine zipper transcription factor, which serves as a crucial molecule in inducible cell defense systems by mediating the expression of oxidative-stress-related genes and regulating the transcription of antioxidants and phase II detoxifying enzymes containing heme oxygenase-1 (HO-1) and NAD(P)H: quinine oxidoreductase-1 (NQO-1).^21–23^ Under normal conditions, Nrf2 binds to the repressor kelch-like ECH-associated protein 1 (Keap1) in the cytoplasm and is subject to ubiquitylation and proteosomal degradation.^24,25^ In response to oxidative stress, Nrf2 moves to the nucleus and binds to the antioxidant response element (ARE), which serves as a promoter sequence.^21,25^ Furthermore, Nrf2 has been a new therapeutic target for demyelination in multiple sclerosis (MS).^26,27^ Therefore, we assumed that the upregulation of the Nrf2 pathway may represent a reasonable strategy for mitigating damage to myelin sheaths in lesions in schizophrenia.

**Fig. 1:**
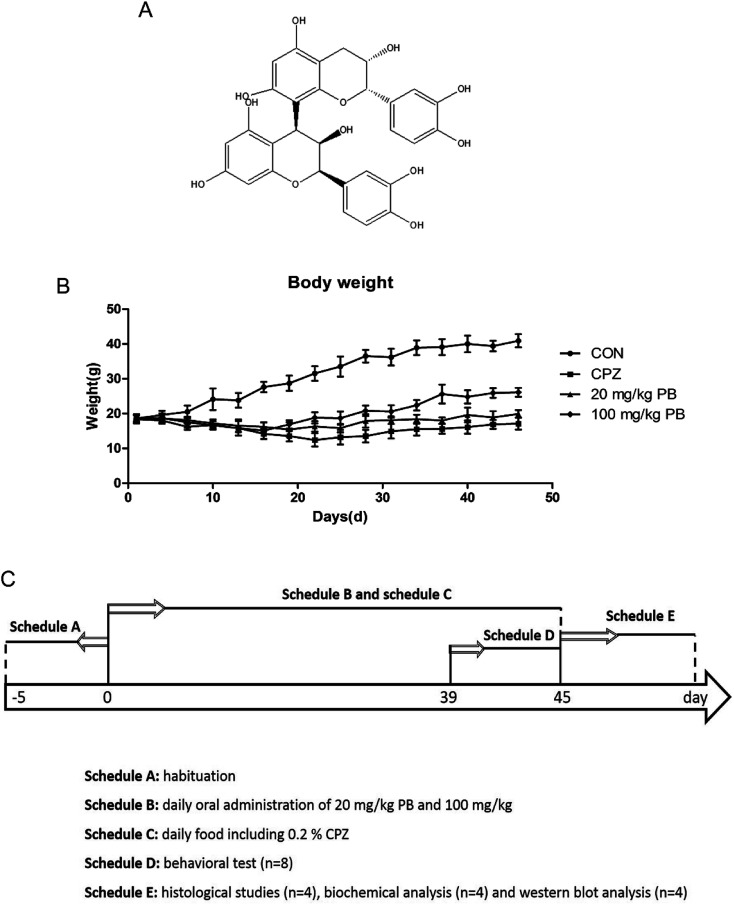
(A) Chemical structure of procyanidin B2. (B) Body weight (*n* = 12) in CON group, CPZ group and PB groups. Two-way ANOVA for body weight indicated a significant group-by-day interaction. (C) Schedule of experimental arrangement including the time course of the administration of cuprizone and procyanidin B2 and timeline for behavioral testing, histological studies and western blotting analysis.

Procyanidins, which are flavanols and plant polyphenols, are abundant in grapes, tea, strawberries, red wine and cocoa products such as chocolate.^28,29^ Procyanidin B2 (PB2) is a dimer derived from two molecules of the flavan-3-ol (−)-epicatechin, and its chemical structure is shown in Fig. 1A. Furthermore, procyanidin B2, which is a representative compound of the flavanol class, serves as a bioactive food component on account of its function and health promotion effect, as well as the restoration and maintenance of homeostasis.^30,31^ In addition, an increasing number of studies illustrated that procyanidin B2 enhances the activities of antioxidant enzymes, as well as protecting against oxidative damage *via* regulating the endogenous cellular defense system.^32,33^ Hence, we suggested that procyanidin B2 may protect against demyelination in schizophrenia by promoting the activities of antioxidant enzymes.

In conclusion, procyanidin B2 provides an effective function against oxidative stress. However, no research has demonstrated whether procyanidin B2 ameliorates locomotor dysfunction and neurological deficits in schizophrenia. In our study, we studied the functional protective effect of procyanidin B2 against demyelination *via* assessing alterations in behavioral and pathological patterns. In addition, we investigated whether the effects of procyanidin B2 are related to the upregulation of the Nrf2 signaling pathway.

## 2. Materials and methods

### 2.1 Animals

Seven-week-old male C57BL/6 mice were purchased from Wuhan University Laboratory Animal Center with body weights ranging between 18 and 20 g. The mice were allowed one week to acclimatize to the new environment under standard experimental conditions with a 12 h/12 h light–dark cycle, a room temperature of 23.0 ± 1.0 °C, a humidity of 60.0 ± 5.0% and enough food and water. Our animal manipulations were carried out according to ARRIVE (Animal Research: Reporting *In Vivo* Experiments) guidelines, and the experiments were allowed by the relevant committees of Wuhan University Center for Animal Experiments, Wuhan, China (AUP no. S20151011I).

### 2.2 Experimental design

Procyanidin B2, which was obtained from Adamas Reagent Co., Ltd (Shanghai, China), was administered in normal saline as a suspension *via* gavage. In addition, its purity was greater than 95% and its melting point is 197–198 °C. The concentrations used in *in vivo* experiments were selected on the basis of a preliminary unpublished study carried out in our laboratory. In the control group the mice were fed with regular chow, but in the other groups the mice were fed with chow containing 0.2% (w/w) cuprizone purchased from Sinopharm Chemical Reagent Co., Ltd (Shanghai, China). The animals were divided into four groups (12 animals per group) according to different treatments as follows: (i) a control group (CON), which was fed with normal chow; (ii) a cuprizone-induced group (CPZ), in which myelin sheaths were damaged in the CNS after a diet of chow including 0.2% (w/w) cuprizone, as previously described in the literature;^12^ (iii) a group treated with 20 mg kg^−1^ procyanidin B2 (20 mg kg^−1^ PB), which were fed with the same chow with cuprizone toxicity, as well as 20 mg kg^−1^ procyanidin B2; and (iv) a group treated with 100 mg kg^−1^ procyanidin B2 (100 mg kg^−1^ PB), which were administered a diet with 100 mg kg^−1^ procyanidin B2 and cuprizone. In addition, body weights were measured every two days at noon to assess the body condition. Moreover, a behavioral experiment was performed during the sixth week, and then the cuprizone-intoxicated mice were euthanized for western blotting analysis and a histopathological study. The laboratory schedule is shown in Fig. 1C.

### 2.3 Open-field test

To assess the motor activity, exploratory behavior and emotional state of animals (*n* = 8), an open-field test was performed after a diet containing cuprizone for five weeks. The experimental environment was sufficiently silent throughout the whole test. The animals (*n* = 8) were placed into an open-field hardboard box for 6 min individually. During the original period, a time of 1 min was allowed for accommodation and 5 min for recording, including both horizontal and vertical movement and dwell time. In addition, the mice were allowed optional movement in the box during the experiment.

### 2.4 Rotarod test

A rotarod test was carried out to assess locomotion, balance and coordination during the fifth week. Animals (*n* = 8) were adapted to an accelerating rotarod at 30 rpm for 300 s on the day before the test. After training, the locomotion time for which mice stayed on the rod (the time at which they first fell from the cylinders) was recorded. If an animal fell from the rod, it was placed back onto the rod, and the number of falls from the cylinder in 300 s was counted, as previously reported.^34^

### 2.5 Morris water maze

As previously reported,^35^ the spatial learning and memory ability of animals (*n* = 8) were estimated *via* a Morris water maze test in a quiet environment. In our study, a galvanized metal tank with a height of 55 cm and a diameter of 160 cm was filled with water, and a platform was located about 1 cm below the surface of the water at room temperature. The experiment comprised two periods, namely, an acquisition stage and a probe stage. Before training and formal testing, mice were placed onto the fixed platform for 30 s to acquaint them with this test. The Morris water maze apparatus was divided into four parts (quadrants I, II, III and IV), and mice (*n* = 8) were placed in different quadrants with their faces to the pool wall. In the acquisition stage, the animals were trained using a sequence of quadrants to become accustomed to swimming in the pool (once for 60 s) and seeking the fixed platform each day, which was performed repetitively for four days. During the first stage, if animals could not find the hidden platform, we would guide them to the platform with our hands. In the probe stage, an animal was placed in the pool as in the first stage without the hidden platform. A camera was installed above the tank, which was also equipped with Morris water maze analysis software (Chengdu Techman Software Co., Ltd) to record the tracks of the mice.

### 2.6 Tissue preparation and myelin staining

After the behavioral tests, mice (*n* = 4) were anaesthetized deeply with chloral hydrate *via* intraperitoneal injection. In addition, animals were perfused transcardially with saline followed by 4% paraformaldehyde (PFA). Then, brains were separated, followed by post-fixation in 4% PFA for 24 h, and blocked and embedded in paraffin. Ultimately, sagittal sections were obtained with a thickness of 8 μm between 1.20 and 1.32 mm from the corpus callosum toward the bregma.^36^

Sections were deparaffinized in fresh xylene for 10 min twice, followed by dehydration with an alcohol gradient (100%, 10 min × 2; 95%, 5 min; 80%, 5 min; 70%, 5 min). Subsequently, demyelination was illustrated by staining paraffin sections with Luxol fast blue (LFB). After immersion and incubation in a 0.01% LFB solution overnight at 60 °C, the slides were rinsed with 95% and 70% ethyl alcohol and distilled water. Sections were immersed and differentiated in a lithium carbonate solution for 30 s and then in 70% ethyl alcohol for a further 30 s. Finally, sections were dehydrated with a gradient series of alcohol, cleared in xylene and mounted.^37^

### 2.7 Immunofluorescence staining for MBP

To estimate the degree of demyelination, we conducted immunofluorescence staining for myelin basic protein (MBP). At room temperature, endogenous peroxidase activity was blocked with 3% H_2_O_2_ in PBS for 10 min after paraffin sections were dewaxed and hydrated. After being washed with PBS, the sections were immersed in 0.01 mol L^−1^ citric acid buffer (pH 6.0) for 20 min to retrieve antigens. Then, 5% bovine serum albumin (BSA) was utilized to block nonspecific binding sites in the sections. Subsequently, the sections were incubated at 4 °C overnight with rabbit anti-MBP (1 : 250, Boster Biotechnology, Wuhan, China) as the primary antibody. After washing with PBS three times, we used a specific goat fluorescein isothiocyanate (FITC)-conjugated secondary antibody (1 : 1000 dilution, Beyotime Institute of Biotechnology) for immunofluorescence staining. In addition, the nuclei were stained with 4′,6-diamidino-2-phenylindole (DAPI, 1 : 500, Beyotime Institute of Biotechnology). Finally, images were acquired using an inverted fluorescence microscope (Olympus, Japan) with a charge-coupled device camera system. The integrated optical density (IOD) of fluorescence microphotographs was estimated and analyzed as previously described using Image-Pro Plus 6.0 software.

### 2.8 Biochemical analysis

Animals (*n* = 4) were euthanized when the behavioral tests were completed, and then brain homogenates (10%, w/v) were obtained with ice-cold potassium phosphate buffer (pH 7.4). The suspensions were centrifuged at 3000 rpm for 10 min at 4 °C, and the supernatants were collected for the measurement of catalase (CAT), malonaldehyde (MDA) and superoxide dismutase (SOD). The activities of these three vital biomarkers (CAT, MDA and SOD) were assayed with commercial reagent kits obtained from the Nanjing Jiangcheng Institute of Biological Engineering (Nanjing, China).

### 2.9 Western blotting analysis

Regions of the corpus callosum were quickly collected from the mouse brains and homogenized in RIPA lysis buffer with the addition of 0.1 mM phenylmethanesulfonyl fluoride as a protease inhibitor. In addition, nuclear and cytoplasmic proteins were extracted using a Nuclear and Cytoplasmic Protein Extraction Kit purchased from Beyotime Biotechnology (Wuhan, China). The concentrations of proteins were measured by a BCA protein assay (Beyotime Biotechnology, Wuhan, China). Then, equivalent amounts of proteins were obtained for western blotting analysis.

Samples of the proteins were loaded onto a 10% sodium dodecyl sulfate-polyacrylamide gel (SDS-PAGE) and subjected to electrophoresis at 80 V and 120 V. Then, the proteins were transferred to polyvinylidene fluoride (PVDF) membranes and incubated with 5% bovine serum albumin (BSA) to block nonspecific binding. Next, the membranes were incubated at 4 °C overnight with primary antibodies against MBP (1 : 250, Boster Biotechnology, Wuhan, China), HO-1 (1 : 500, Boster Biotechnology, Wuhan, China), NQO1 (1 : 1000, Proteintech Group, Inc., Chicago, USA), Nrf2 (1 : 500, Boster Biotechnology, Wuhan, China) and β-actin (1 : 5000, Proteintech Group, Inc., Chicago, USA). Furthermore, the expression of Nrf2 in the nucleus was investigated *via* incubation with anti-Nrf2 and anti-lamin B antibodies^38^ (1 : 200, Boster Biotechnology, Wuhan, China). After rinsing with Tris-buffered saline containing Tween-20 (TBST), the blots were incubated using a secondary antibody (1 : 10 000, Santa Cruz Biotechnology, CA, USA) conjugated to horseradish peroxidase. Finally, chemiluminescence reagents (Goodbio Technology, Wuhan, China) were used to perform a chemiluminescence autographic assay and quantify the relative expression. Data analysis was performed with ImageJ software.

### 2.10 Statistical analysis

All data were expressed as the mean ± standard error of the mean (SEM). One- and two-way analyses of variance (ANOVA) were performed to compare the experimental and control groups. Statistical evaluation and graphs of the results were produced using GraphPad Prism 5. A difference was considered to be significant if the value of *p* was <0.05.

## 3. Results

### 3.1 Procyanidin B2 increases the body weight of mice

The body weights of the mice were measured and recorded every two days, and the results are shown in Fig. 1B. The weight of the mice in the CON group rose rapidly from 18.6 ± 1.2 g to 40.9 ± 1.9 g, which represented a growth rate of 119.9%. In the CPZ group, the weight of the animals underwent a decline of 4.3%, decreasing from 18.5 ± 1.3 g to 17.1 ± 1.7 g with statistical significance (*p* < 0.05) in comparison with the CON group. As shown in Fig. 1B, the weight of the mice in the 20 mg kg^−1^ PB group increased by about 6.5% from 18.7 ± 0.9 g to 19.9 ± 1.1 g. In the 100 mg kg^−1^ PB group, the weight of the mice underwent an increase of 41.8%, rising from 18.4 ± 1.1 g to 26.1 ± 1.3 g, which represented a difference in comparison with the CPZ group (*p* < 0.05). Fig. 1B indicated that cuprizone toxicity induced an enormous reduction in mouse body weight in contrast to the control animals. Procyanidin B2 prevented decreases in mouse body weight induced by cuprizone, especially in mice fed with 100 mg kg^−1^ procyanidin B2. The results indicated that procyanidin B2 was beneficial for increasing the body weight of mice fed with cuprizone.

### 3.2 Procyanidin B2 enhances exploratory activity and motor coordination in animals

**Fig. 2:**
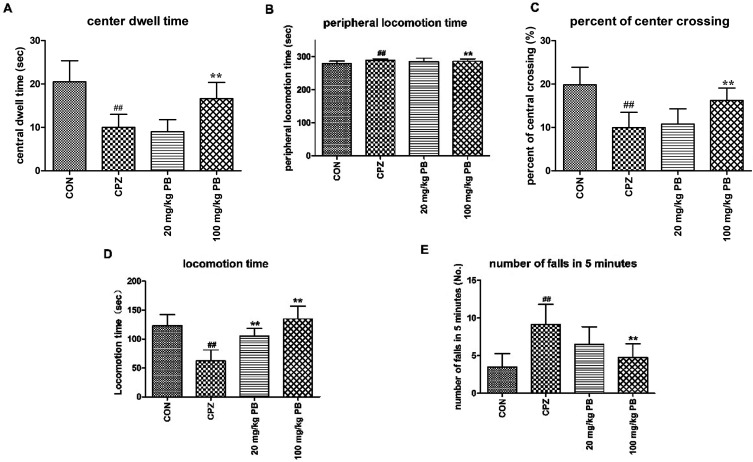
The locomotor influence of cuprizone intoxication and treatment with procyanidin B2 was assessed. (A–C) Open-field test. The center dwell time (A), peripheral locomotion time (B) and center crossing percentage (C) were recorded. Cuprizone-intoxicated mice spent shorter times and covered less distance in the central grids, which was significantly reversed by treatment with 100 mg kg^−1^ procyanidin B2 (*p* < 0.01). (D and E) Rotarod test. Cuprizone-intoxicated mice stayed for the shortest time and had the greatest number of falls from the rotating cylinder, which was ameliorated by procyanidin B2, with a significant difference (*p* < 0.01). All data are expressed as the mean ± SEM (*n* = 8). ^#^*p* < 0.05, ^##^*p* < 0.01 *versus* the CON group; *p < 0.05, **p < 0.01 *versus* the CPZ group.

Open-field tests were performed to assess the initiative and motor behavior of animals after being fed with cuprizone for five weeks. Mice that were poisoned by cuprizone tended to exhibit immobility rather than moving around the box to explore the outside world. As Fig. 2A–C shows, the control animals were active with a center dwell time of 25 ± 4.8 s and a center crossing percentage of 19.8%. Evidently, the mice in the CPZ group rarely crossed into the center of the field, with a center dwell time of 13 ± 3.1 s and a center crossing percentage of 9.9% in comparison with the CON group. After treatment with procyanidin B2, the center crossing percentage underwent increases of 10.8% and 16.3%, respectively, and the 100 mg kg^−1^ PB group exhibited a significant difference (*p* < 0.01). In addition, the center dwell time was not prolonged by 20 mg kg^−1^ procyanidin B2 (9 ± 2.8 s), but the mice in the 100 mg kg^−1^ PB group moved for longer times in the center of the field (16 ± 3.7 s), with a significant statistical difference (*p* < 0.01). Hence, 100 mg kg^−1^ procyanidin B2 could reverse the loss of locomotor activity induced by cuprizone efficiently (Fig. 2A–C).

To appraise the motor coordination and balance ability of the animals, a rotarod test was conducted after feeding with cuprizone for five weeks. The locomotion time on the high-speed rotating cylinder and the number of falls were recorded to estimate motor and balance activity. As Fig. 2D shows, the mice treated with 100 mg kg^−1^ procyanidin B2 remained for the longest time on the cylinder (134.9 ± 22.1 s) among the four groups, with a marked difference (*p* < 0.01). In particular, the mice in the 100 mg kg^−1^ PB group (4.8 ± 1.8) ranked only second to the CON group (3.5 ± 1.8) in terms of the number of falls in 5 minutes, with a significant difference (*p* < 0.01). Mice that were poisoned by cuprizone exhibited the shortest locomotion time (123.3 ± 18.8 s) on the high-speed cylinder and the maximum number of falls (9.1 ± 2.7) from the cylinder in 5 minutes, with statistical significance (*p* < 0.01). The mice in the 20 mg kg^−1^ PB group displayed improvements in balance and motor activity by remaining on the cylinder for 105.4 ± 12.9 s, with a statistically significant difference (*p* < 0.01) in comparison with the CPZ group. In brief, animals that were poisoned by cuprizone tended to fall down in comparison with the control mice. However, the administration of procyanidin B2 to mice reversed damage to motor coordination caused by cuprizone.

### 3.3 Procyanidin B2 restores spatial memory and cognition impaired by cuprizone

**Fig. 3:**
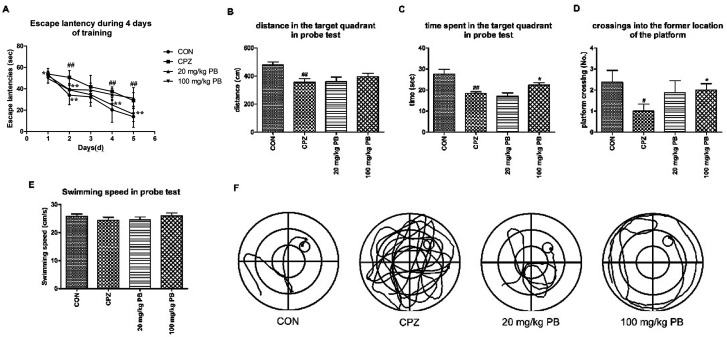
Effect of procyanidin B2 on cuprizone-induced impairments in spatial cognition and memory assessed by the MWM task. (A) Two-way ANOVA for escape latencies shown as the mean value for each training session for four successive days. The distance covered (B) and time spent (C) in the target quadrant were measured during the task. Cuprizone increased the number of crossings (D) to the location of the removed platform, with a significant difference (*p* < 0.01), which was increased by procyanidin B2 (*p* < 0.05). In addition, procyanidin B2 increased the swimming speed of mice in the probe test (E) in comparison with mice in the CPZ group. Swimming tracks (F) were recorded *via* a video tracking camera system. All data are expressed as the mean ± SEM (*n* = 8). ^#^*p* < 0.05, ^##^*p* < 0.01 *versus* the CON group; *p < 0.05 *versus* the CPZ group.

After five weeks, MWM tests were performed to estimate the effect of PB on damage to spatial memory and cognition. As Fig. 3A shows, the mice in the CON group learned most rapidly and the mice in the CPZ group learned most slowly among the four groups. The mice treated with 100 mg kg^−1^ procyanidin B2 learned to find the platform quickly and ranked only second to the control animals, with a statistically significant difference (*p* < 0.01). Evidence demonstrated that PB could alleviate damage to spatial memory in mice caused by cuprizone according to the value of the escape latency. In the probe test, we discovered that damage to spatial cognition in mice could be alleviated by feeding with 100 mg kg^−1^ PB, according to Fig. 3B–D. The animals in the CPZ group exhibited a significant difference (*p* < 0.01) in traveled distance and time spent in the target quadrant in the probe test and crossed to the former location of the platform less than the control animals, with a significant difference (*p* < 0.05). However, after the administration of procyanidin B2 the mice moved longer distances and spent more time in the target quadrant. Certainly, the mice in the 100 mg kg^−1^ PB group crossed to the former location of the platform more than the mice in the CPZ group, with a significant difference (*p* < 0.05). In addition, the mice in the CON (25.9 ± 2.2 cm s^−1^) and 100 mg kg^−1^ PB (26.0 ± 2.7 cm s^−1^) groups moved more rapidly than the mice in the CPZ (24.5 ± 2.9 cm s^−1^) and 20 mg kg^−1^ PB (24.6 ± 3.1 cm s^−1^) groups (Fig. 3E). The statistics indicated that cuprizone intoxication leads to damage to spatial learning and memory in mice, which could be alleviated by treatment with 100 mg kg^−1^ procyanidin B2 (Fig. 3F).

### 3.4 Procyanidin B2 blocks cuprizone-induced demyelination

**Fig. 4:**
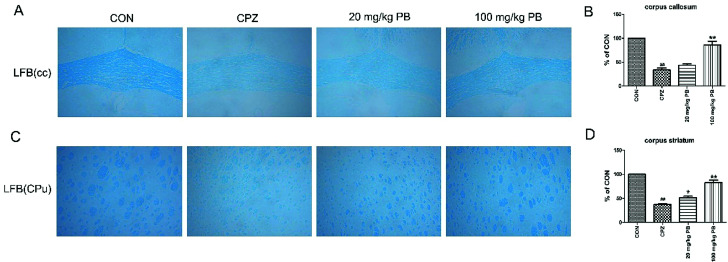
Protective effect of procyanidin B2 on white matter against demyelinated lesions revealed in the corpus callosum (cc) and corpus striatum (CPu) by LFB staining. LFB staining (A and C) showed the severity of demyelination in the cc and CPu. The blue areas represent intact regions, and data are given as quantified for LFB staining (B and D). All data are expressed as the mean ± SEM (*n* = 4). ^##^*p* < 0.01 *versus* the CON group; *p < 0.05, **p < 0.01 *versus* the CPZ group.

The degree of demyelination in the corpus callosum and caudoputamen was assessed by LFB staining (Fig. 4A and C). LBF staining indicates the integrity of myelin sheaths, as well as revealing fibrosis and structural injury to myelin to show pathological disruption of white matter.^39^ An entire organized structure of myelin was revealed in the CON group, but LFB staining in the CPZ group indicated the destruction of myelin sheaths, with a significant difference (*p* < 0.01). As Fig. 4 shows, the administration of procyanidin B2 could improve the structure of myelin; in particular, treatment with 100 mg kg^−1^ procyanidin B2, with a significant difference (*p* < 0.01). The results indicated that the administration of 100 mg kg^−1^ procyanidin B2 could reverse demyelination caused by cuprizone toxicity.

### 3.5 Procyanidin B2 facilitates the expression of MBP

**Fig. 5:**
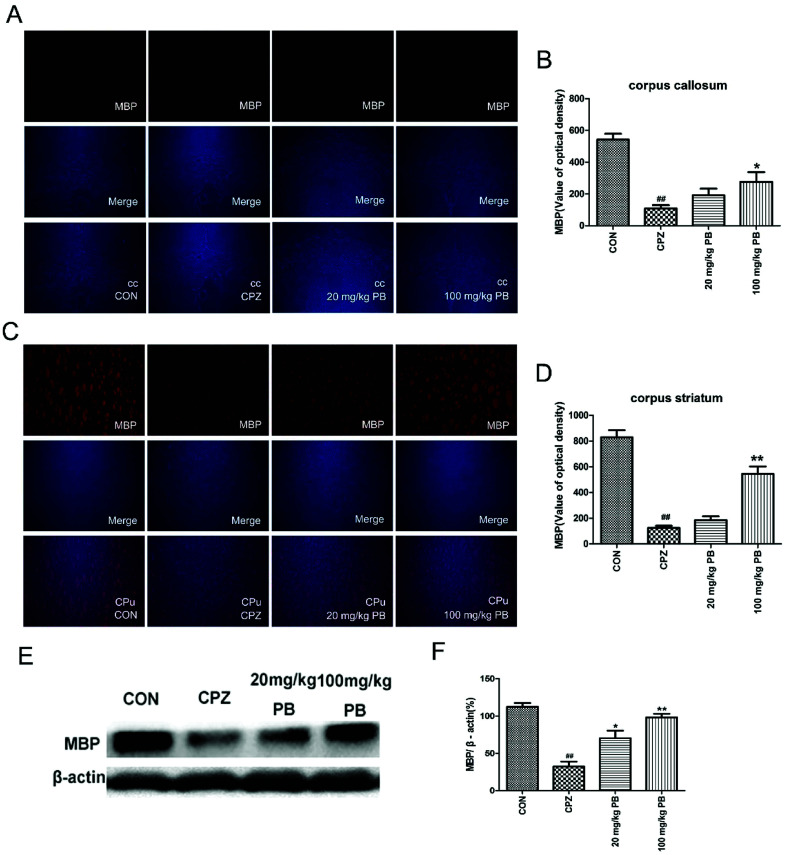
Immunofluorescence staining of MBP in the cc (A) and CPu (C) was performed, and merged images are shown. The immunofluorescence optical density of MBP (B and D) demonstrated that 100 mg kg^−1^ procyanidin B2 evidently alleviated demyelination caused by cuprizone. The effect of procyanidin B2 on myelin proteins in cuprizone-intoxicated mice was analyzed by western blotting (E and F). The densified values (F) indicated that the reduction in the expression of MBP in the cc and CPu of cuprizone-intoxicated mice was significantly reversed by procyanidin B2. All data are expressed as the mean ± SEM (*n* = 4). ^##^*p* < 0.01 *versus* the CON group; **p < 0.01 *versus* the CPZ group.

To determine the expression of the MBP protein in the corpus callosum and caudoputamen, immunofluorescence and western blotting analyses of MBP were performed. Immunofluorescence images (Fig. 5A and C) demonstrated that cuprizone intoxication could greatly decrease the quantity of MBP, with a significant difference (*p* < 0.01). As Fig. 5B and D show, the fluorescence intensity was enormously enhanced by the administration of 100 mg kg^−1^ procyanidin B2. In brief, the reduction in the quantity of MBP was alleviated *via* the administration of 100 mg kg^−1^ procyanidin B2, which indicated that procyanidin B2 might prevent myelin sheaths from being completely demyelinated. Western blotting analysis of MBP determined the MBP protein expression level to reflect the extent of myelination and demyelination in the animals. In comparison with the CON group, the MBP expression level underwent a massive reduction after cuprizone intoxication. The administration of 100 mg kg^−1^ procyanidin B2 could increase the expression of MBP dramatically in comparison with the CPZ group. Fig. 5E and F revealed that treatment with procyanidin B2 could markedly improve the distribution and expression of MBP.

### 3.6 PB regulates oxidative stress and promotes the expression of phase II detoxifying enzymes

**Fig. 6:**
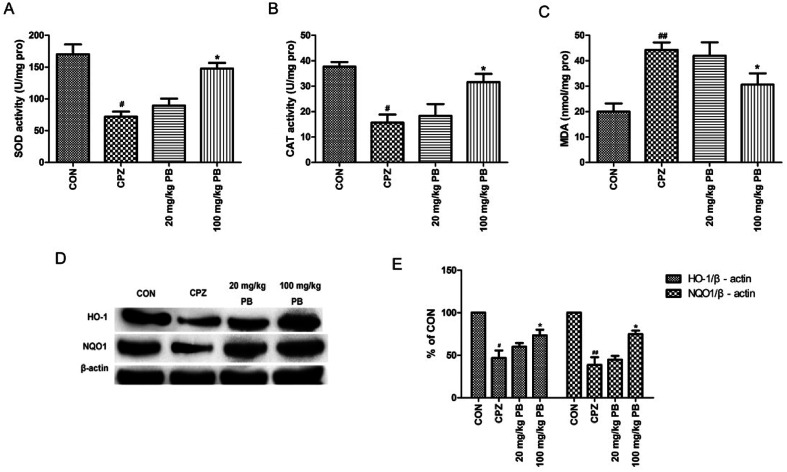
The influence of PB on oxidative stress and phase II detoxifying enzymes was researched. The SOD activities (A), CAT activities (B) and MDA levels (C) in the experiments are shown. Representative western blots (D) for the expression of HO-1 and NQO1 in the CON, CPZ, 20 mg kg^−1^ PB and 100 mg kg^−1^ PB groups were evaluated. The densified values (E) indicated that procyanidin B2 facilitated recovery of the expression of HO-1 and NQO1. All data are expressed as the mean ± SEM (*n* = 4). ^#^*p* < 0.05, ^##^*p* < 0.01 *versus* the CON group; *p < 0.05 *versus* the CPZ group.

MDA levels and the activities of enzymes relevant to oxidative stress, including SOD and CAT, were measured to study the effect of PB on oxidative stress. According to Fig. 6A–C, we found that levels of MDA were reduced and the activities of SOD and CAT were enhanced by administering 100 mg kg^−1^ procyanidin B2, with a significant difference (*p* < 0.05). To assess the expressions of phase II detoxification proteins, western blotting analyses were performed. Expression levels of HO-1 and NQO1 were measured using western blotting analysis, as shown in Fig. 6D. The protein expression of HO-1 and NQO1 decreased significantly after cuprizone intoxication (Fig. 6E). The amount of expressed protein increased after treatment with 100 mg kg^−1^ procyanidin B2, with a significant difference (*p* < 0.05). According to the results, we found that procyanidin B2 could upregulate the expression of phase II detoxifying enzymes, including SOD and CAT.

### 3.7 PB potentially modulates Nrf2 expression

**Fig. 7:**
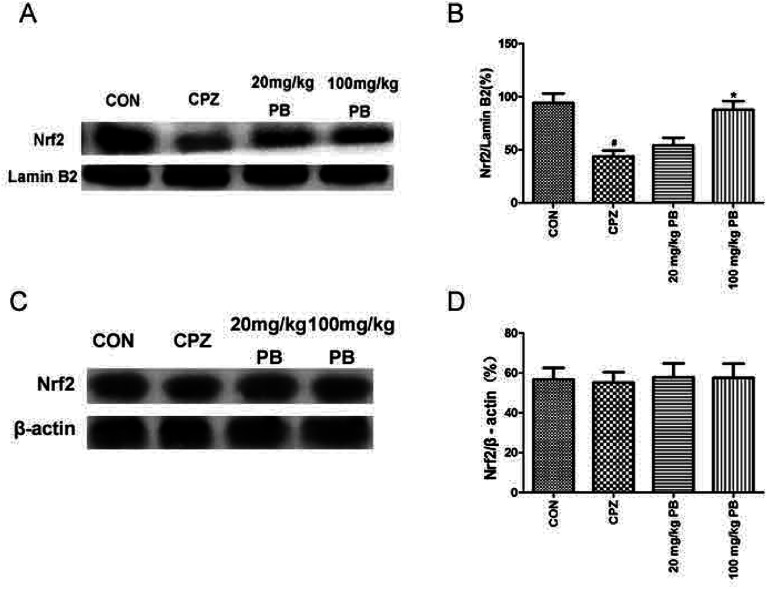
The effects of cuprizone and PB on the protein expression of Nrf2 in the nuclear and cytosolic fractions were studied using western blotting analysis. Representative western blots (A) revealed that the expression of Nrf2 in the nuclear fraction was downregulated by cuprizone intoxication. Densified value (B) of Nrf2 expression normalized to that of lamin B2. Representative western blots (C) show that the expression of Nrf2 remained unchanged. Densified value (D) of Nrf2 expression normalized to that of β-actin. All data are expressed as the mean ± SEM (*n* = 4). ^#^*p* < 0.05 *versus* the CON group; *p < 0.05 *versus* the CPZ group.

We studied whether the function of procyanidin B2 is relevant to Nrf2 signaling pathways *via* the upregulation of Nrf2. Then, we used western blotting analysis to determine the expression of Nrf2 protein in the nuclear and cytosolic fractions. The amount of Nrf2 that moved from the cytoplasm to the nucleus was estimated using the expression of Nrf2 protein in the nuclear fraction. The expression of Nrf2 protein in the cytosolic fraction was within the same range (Fig. 7A and B). In the nuclear fraction, Nrf2 translocation was inhibited after feeding with cuprizone for five weeks (Fig. 7C and D). However, the administration of procyanidin B2 could compete with cuprizone intoxication. In brief, treatment with procyanidin B2 may activate the Nrf2 signaling pathway by promoting the nuclear translocation of Nrf2.

## 4. Discussion

It is a topical issue whether damage to white matter in the brain in schizophrenia is related to oxidative stress by inhibiting oxidation–reduction reactions. For the first time, we studied the function of procyanidin B2 in schizophrenia induced by cuprizone toxicity. Our data indicate that procyanidin B2 ameliorated behavioral deficits, including those in exploration, motor coordination and spatial cognition. In addition, procyanidin B2 could reduce the degree of damage due to demyelination. The focus of this study was whether: (i) procyanidin B2 could effectively promote the restoration of motor dysfunction caused by cuprizone; (ii) procyanidin B2 could sufficiently protect myelin sheaths from damage due to oxidative stress; and (iii) the improvements due to procyanidin B2 are correlated with the activation of the Nrf2 signaling pathway.

Schizophrenia is one of the most severe mental disorders. Much evidence suggests that not only abnormalities in grey matter, but also abnormalities in white matter were detected by diffusion tensor imaging (DTI) in patients with schizophrenia.^40^ In patients with schizophrenic brains, gene expression analysis indicated the downregulation of oligodendrocyte- and myelin-related genes.^41,42^ Oligodendrocyte restoration and myelin repair played a critical role in the treatment of schizophrenia.^43^ Previous reports suggested that cuprizone damages mature oligodendrocytes and leads to selective demyelination.^44^ Cuprizone-exposed mice and rats exhibit abnormal behavior including motor coordination deficits, motor dysfunction, impairments in social interaction and cognitive damage.^34,45^ In addition, the synthesis of myelin sheaths requires a high metabolic demand, with the result that oligodendrocytes are vulnerable to oxidative stress.^46,47^ With the administration of cuprizone, levels of reactive radicals were elevated and the brain was damaged because of the production of reactive oxygen species.^48^ Evidence showed that the activities of antioxidant enzymes including GPx and SOD were reduced *via* cuprizone intoxication.^16^ In this study, we found that the activities of SOD and CAT rose and the level of MDA was reduced by a diet including cuprizone. However, procyanidin B2 alleviated oxidative stress to protect myelin from cuprizone toxicity.

Increasing evidence has shown that the upregulation of Nrf2 gene expression is beneficial for protecting cells or animals from oxidative stress.^49^ Nrf2 had a strong protective function against oxidative stress and inflammation.^50,51^ The activation of Nrf2 signaling pathways had neuroprotective effects in models of central nervous system diseases including ischemic cerebral injury, experimental autoimmune encephalomyelitis, Alzheimer's disease (AD) and so on.^52–54^ In an animal model of cuprizone-induced multiple sclerosis, Nrf2 activation was sufficient to prevent the loss of oligodendrocytes and demyelination.^26^ In addition, Nrf2 played a critical role in the treatment of phencyclidine-induced abnormal behavior in mice.^55^ In our study, we demonstrated that procyanidin B2 could upregulate the expression of the Nrf2-regulated enzymes HO-1 and NQO1. Therefore, procyanidin B2 may increase the levels of antioxidants and the activities of phase II detoxifying enzymes *via* activating the Nrf2 pathway, which thereby protects against cuprizone-induced oxidative damage.

Procyanidin B2, which is a dimer that consists of the flavanol (−)-epicatechin, has potential antioxidant activities for the prevention and treatment of inflammatory diseases. Numerous studies have indicated that procyanidin B2 may be a promising candidate as an antineoplastic drug by modulating a signal transduction pathway, as well as owing to its anti-inflammatory properties.^56–58^ It has previously been reported that procyanidin B2 possesses potent antioxidant and pro-oxidant activities, as well as a critical role in cell apoptosis caused by oxidative stress.^59,60^ Procyanidin B2 could protect human colonic cells against oxidative injury by upregulating the translocation of Nrf2.^33^ In addition, it has been reported that procyanidin B2 alleviated neurological deficits and disruption of the blood-brain barrier in a model of cerebral ischemia.^61^ We found that procyanidin B2 mitigated schizophrenia-like behavioral impairment in a cuprizone-induced mouse model, as well as upregulating the expression of Nrf2 protein. Hence, procyanidin B2 may be a potential agent for treating schizophrenia *via* activating the Nrf2 signaling pathway to protect myelin from oxidative stress. As has been reported,^62^ procyanidin B2 is absorbed and excreted in the urine after administration. Furthermore, a portion of procyanidin B2 is degraded to (−)-epicatechin and metabolized conjugated and/or methylated (−)-epicatechin internally in the rat. Our study has indicated that procyanidin B2 may be a potential therapeutic agent for schizophrenia. Moreover, we are trying to determine the suitable dosage of procyanidin B2, which would be a promising nutritional supplement for protecting myelin sheaths.

At present, patients with schizophrenia impose a severe social burden, and the pathophysiology of schizophrenia remains poorly understood.^63^ In patients with chronic schizophrenia, oxidative stress was proposed as an etiological factor and a potential target in the treatment of psychiatric disorders including depression, anxiety, autism and schizophrenia.^64,65^ In brief, oxidative stress has a close relationship with the treatment of schizophrenia. Reports indicated that antioxidant activity and the activation of the Nrf2 pathway were hopeful as promising therapeutic strategies for the administration of neuroprotective agents.^66,67^ When the Nrf2 signaling pathway was activated, a cysteine residue in Keap1 was modified with a covalent adduct or disulfide bonds within the protein, and ARE was upregulated. ARE facilitated the activities of phase II antioxidant enzymes including HO-1 and NQO1, which are related to the Nrf2 pathway. In our study, we found that the expression of HO-1 and NQO1 was upregulated and the nuclear translocation of Nrf2 was accelerated after treatment with procyanidin B2. Moreover, procyanidin B2 could protect mice against damage due to oxidative stress *via* upregulating the Nrf2 signaling pathway.

## 5. Conclusion

In conclusion, statistics suggested that procyanidin B2 mitigates schizophrenia-like behavioral impairments involving exploration, motor coordination and spatial cognition and protects myelin integrity in cuprizone-intoxicated mice. In addition, procyanidin B2 may alleviate behavioral dysfunction caused by cuprizone toxicity *via* upregulating the Nrf2 signaling pathway to compete with oxidative stress. Furthermore, our experimental data revealed that procyanidin B2 may be a potential myelin-protective agent in schizophrenia treatment.

## Conflicts of interest

There are no conflicts to declare.

## Supplementary Material

RA-008-C8RA03854F-s001

## References

[cit1] Corfas G., Roy K., Buxbaum J. D. (2004). Nat. Neurosci..

[cit2] Cheng H., Newman S. D., Kent J. S., Bolbecker A., Klaunig M. J., O'Donnell B. F., Puce A., Hetrick W. P. (2015). Brain imaging and behavior..

[cit3] Krabbendam L., Arts B., van Os J., Aleman A. (2005). Schizophr. Res..

[cit4] Zhu J., Zhuo C., Qin W., Wang D., Ma X., Zhou Y., Yu C. (2015). Neuroimage Clin..

[cit5] Kikinis Z., Cho K. I., Coman I. L., Radoeva P. D., Bouix S., Tang Y., Eckbo R., Makris N., Kwon J. S., Kubicki M., Antshel K. M., Fremont W., Shenton M. E., Kates W. R. (2016). Brain imaging and behavior..

[cit6] Tomlinson L., Leiton C. V., Colognato H. (2016). Neuropharmacology.

[cit7] Wright S. N., Kochunov P., Chiappelli J., McMahon R. P., Muellerklein F., Wijtenburg S. A., White M. G., Rowland L. M., Hong L. E. (2014). Neurobiol. Aging..

[cit8] Lei W., Li N., Deng W., Li M., Huang C., Ma X., Wang Q., Guo W., Li Y., Jiang L., Zhou Y., Hu X., McAlonan G. M., Li T. (2015). Sci. Rep..

[cit9] Radu A., Hristescu G., Katsel P., Haroutunian V., Davis K. L. (2011). Adv. Exp. Med. Biol..

[cit10] Messori L., Casini A., Gabbiani C., Sorace L., Muniz-Miranda M., Zatta P. (2007). Dalton Trans..

[cit11] Ludwin S. K. (1978). Lab. Invest..

[cit12] Herring N. R., Konradi C. (2011). Front. Biosci..

[cit13] Xu H., Yang H. J., McConomy B., Browning R., Li X. M. (2010). Front. Behav. Neurosci..

[cit14] Wang H. N., Liu G. H., Zhang R. G., Xue F., Wu D., Chen Y. C., Peng Y., Peng Z. W., Tan Q. R. (2015). Int. J. Neuropsychopharmacol..

[cit15] Faizi M., Salimi A., Seydi E., Naserzadeh P., Kouhnavard M., Rahimi A., Pourahmad J. (2016). Toxicol. Mech. Methods.

[cit16] Xuan Y., Yan G., Wu R., Huang Q., Li X., Xu H. (2015). Neurochem. Int..

[cit17] Ciobica A., Padurariu M., Dobrin I., Stefanescu C., Dobrin R. (2011). Psychiatr. Danubina.

[cit18] Tuncel O. K., Sarisoy G., Bilgici B., Pazvantoglu O., Cetin E., Unverdi E., Avci B., Boke O. (2015). Psychiatry Res..

[cit19] Prabakaran S., Swatton J. E., Ryan M. M., Huffaker S. J., Huang J. T., Griffin J. L., Wayland M., Freeman T., Dudbridge F., Lilley K. S., Karp N. A., Hester S., Tkachev D., Mimmack M. L., Yolken R. H., Webster M. J., Torrey E. F., Bahn S. (2004). Mol. Psychiatry.

[cit20] Liu J., Tian D., Murugan M., Eyo U. B., Dreyfus C. F., Wang W., Wu L. J. (2015). J. Neurochem..

[cit21] Buendia I., Michalska P., Navarro E., Gameiro I., Egea J., Leon R. (2016). Pharmacol. Ther..

[cit22] Xue D., Zhou C., Shi Y., Lu H., Xu R., He X. (2016). Oncotarget.

[cit23] Choi Y. H. (2016). Gen. Physiol. Biophys..

[cit24] Canning P., Sorrell F. J., Bullock A. N. (2015). Free Radic. Biol. Med.

[cit25] Qin S., Hou D. X. (2016). Mol. Nutr. Food Res..

[cit26] Draheim T., Liessem A., Scheld M., Wilms F., Weissflog M., Denecke B., Kensler T. W., Zendedel A., Beyer C., Kipp M., Wruck C. J., Fragoulis A., Clarner T. (2016). Glia.

[cit27] Licht-Mayer S., Wimmer I., Traffehn S., Metz I., Bruck W., Bauer J., Bradl M., Lassmann H. (2015). Acta. Neuropathol..

[cit28] Stoupi S., Williamson G., Viton F., Barron D., King L. J., Brown J. E., Clifford M. N. (2010). Drug Metab. Dispos..

[cit29] Liszt K. I., Eder R., Wendelin S., Somoza V. (2015). J. Agric. Food Chem..

[cit30] Osakabe N. (2013). J. Clin. Biochem. Nutr..

[cit31] Martinez-Micaelo N., Gonzalez-Abuin N., Pinent M., Ardevol A., Blay M. (2015). Mol. Nutr. Food Res..

[cit32] Yang B. Y., Zhang X. Y., Guan S. W., Hua Z. C. (2015). Molecules.

[cit33] Rodriguez-Ramiro I., Ramos S., Bravo L., Goya L., Martin M. A. (2012). Eur. J. Nutr..

[cit34] Iwasa K., Yamamoto S., Takahashi M., Suzuki S., Yagishita S., Awaji T., Maruyama K., Yoshikawa K. (2014). Prostaglandins, Leukotrienes Essent..

[cit35] Zhang Q., Li Z., Wu S., Li X., Sang Y., Li J., Niu Y., Ding H. (2016). Food Funct..

[cit36] Makinodan M., Yamauchi T., Tatsumi K., Okuda H., Takeda T., Kiuchi K., Sadamatsu M., Wanaka A., Kishimoto T. (2009). Prog. Neuro-Psychopharmacol..

[cit37] Vakilzadeh G., Khodagholi F., Ghadiri T., Ghaemi A., Noorbakhsh F., Sharifzadeh M., Gorji A. (2016). Mol. Neurobiol..

[cit38] Kaspar J. W., Jaiswal A. K. (2011). FASEB J..

[cit39] Marcol W., Slusarczyk W., Gzik M., Larysz-Brysz M., Bobrowski M., Grynkiewicz-Bylina B., Rosicka P., Kalita K., Weglarz W., Barski J. J., Kotulska K., Labuzek K., Lewin-Kowalik J. (2012). J. Reconstr. Microsurg.

[cit40] Douaud G., Smith S., Jenkinson M., Behrens T., Johansen-Berg H., Vickers J., James S., Voets N., Watkins K., Matthews P. M., James A. (2007). Brain.

[cit41] Hakak Y., Walker J. R., Li C., Wong W. H., Davis K. L., Buxbaum J. D., Haroutunian V., Fienberg A. A. (2001). Proc. Natl. Acad. Sci. U. S. A..

[cit42] Katsel P., Davis K. L., Haroutunian V. (2005). Schizophr Res..

[cit43] Franklin R. J., Ffrench-Constant C. (2008). Nat. Rev. Neurosci..

[cit44] Hoyos H. C., Marder M., Ulrich R., Gudi V., Stangel M., Rabinovich G. A., Pasquini L. A., Pasquini J. M. (2016). Adv. Exp. Med. Biol..

[cit45] Zimmermann J., Emrich M., Krauthausen M., Saxe S., Nitsch L., Heneka M. T., Campbell I. L., Muller M. (2017). Mol. Neurobiol..

[cit46] Skripuletz T., Gudi V., Hackstette D., Stangel M. (2011). Histol. Histopathol..

[cit47] Acs P., Selak M. A., Komoly S., Kalman B. (2013). J. Neuroimmunol..

[cit48] Ozdemir D., Uysal N., Gonenc S., Acikgoz O., Sonmez A., Topcu A., Ozdemir N., Duman M., Semin I., Ozkan H. (2005). Physiol. Res..

[cit49] Song D., Cheng Y., Li X., Wang F., Lu Z., Xiao X., Wang Y. (2017). ACS Appl. Mater. Interfaces.

[cit50] Nallasamy P., Si H., Babu P. V., Pan D., Fu Y., Brooke E. A., Shah H., Zhen W., Zhu H., Liu D., Li Y., Jia Z. (2014). J. Nutr. Biochem..

[cit51] Li B., Kim D. S., Yadav R. K., Kim H. R., Chae H. J. (2015). Int. J. Mol. Med..

[cit52] Larabee C. M., Desai S., Agasing A., Georgescu C., Wren J. D., Axtell R. C., Plafker S. M. (2016). Mol. Vision.

[cit53] Wu G., Zhu L., Yuan X., Chen H., Xiong R., Zhang S., Cheng H., Shen Y., An H., Li T., Li H., Zhang W. (2017). Antioxid. Redox Signaling.

[cit54] Gameiro I., Michalska P., Tenti G., Cores A., Buendia I., Rojo A. I., Georgakopoulos N. D., Hernandez-Guijo J. M., Teresa Ramos M., Wells G., Lopez M. G., Cuadrado A., Menendez J. C., Leon R. (2017). Sci. Rep..

[cit55] Tran T. V., Shin E. J., Jeong J. H., Lee J. W., Lee Y., Jang C. G., Nah S. Y., Lei X. G., Toriumi K., Yamada K., Nabeshima T., Kim H. C. (2016). Mol. Neurobiol..

[cit56] Zhang W. Y., Liu H. Q., Xie K. Q., Yin L. L., Li Y., Kwik-Uribe C. L., Zhu X. Z. (2006). Biochem. Biophys. Res. Commun..

[cit57] Avelar M. M., Gouvea C. M. (2012). Indian J. Pharm. Sci..

[cit58] Shilpi A., Parbin S., Sengupta D., Kar S., Deb M., Rath S. K., Pradhan N., Rakshit M., Patra S. K. (2015). Chem.-Biol. Interact..

[cit59] Sakano K., Mizutani M., Murata M., Oikawa S., Hiraku Y., Kawanishi S. (2005). Free Radic. Biol. Med.

[cit60] Zhang J. Q., Gao B. W., Wang J., Ren Q. L., Chen J. F., Ma Q., Zhang Z. J., Xing B. S. (2016). Oxid. Med. Cell. Longevity.

[cit61] Wu S., Yue Y., Li J., Li Z., Li X., Niu Y., Xiang J., Ding H. (2015). Mol. Nutr. Food Res..

[cit62] Baba S., Osakabe N., Natsume M., Terao J. (2002). Free Radic. Biol. Med.

[cit63] Kumar A., Yadav M., Parle M., Dhingra S., Dhull D. K. (2017). Inflammopharmacology.

[cit64] Gonzalez-Liencres C., Tas C., Brown E. C., Erdin S., Onur E., Cubukcoglu Z., Aydemir O., Esen-Danaci A., Brune M. (2014). BMC Psychiatry.

[cit65] Smaga I., Niedzielska E., Gawlik M., Moniczewski A., Krzek J., Przegalinski E., Pera J., Filip M. (2015). Pharmacol. Rep..

[cit66] Wu G., Liu Z. (2016). Med. Sci. Monit..

[cit67] Liu Z., Wang H., Shi X., Li L., Zhou M., Ding H., Yang Y., Li X., Ding K. (2017). Neurochem. Res..

