# Supplementary material for: Procyanidin B2 mitigates behavioral impairment and protects myelin integrity in cuprizone-induced schizophrenia in mice
Source: RSC Adv. 2018 Jun 29;8(42):23835–46. doi: 10.1039/c8ra03854f (PMC9081829; doi:10.1039/c8ra03854f)
Supplement: RA-008-C8RA03854F-s001 [file RA-008-C8RA03854F-s001.pdf]

Electronic Supplementary Material (ESI) for RSC Advances.
This journal is © The Royal Society of Chemistry 2018

1 Open-field test

1-1 Central dwall time

Number	CON	CPZ	20 mg/kg PB	100 mg/kg PB
1	25	13	9	16
2	18	10	11	23
3	16	8	7	21
4	25	7	7	12
5	13	5	14	13
6	18	12	5	15
7	26	13	9	17
8	23	12	10	16

1-2 Peripheral locomotion time

Number	CON	CPZ	20 mg/kg PB	100 mg/kg PB
1	275	287	271	274
2	282	290	279	287
3	274	292	293	279
4	275	283	293	298
5	287	285	296	287
6	292	288	295	285
7	274	297	281	293
8	277	288	270	284

1-3 Percent of central crossing

Number	CON	CPZ	20 mg/kg PB	100 mg/kg PB
1	19.64	12.64	6.67	16.15
2	22.74	10.64	10.24	20.08
3	25.74	10.59	12.32	18.24
4	13.40	10.92	4.71	13.11
5	15.73	12.00	13.56	11.21
6	18.62	7.69	11.11	17.31
7	23.33	2.25	14.81	17.62
8	19.40	12.99	12.90	16.24

2 Rpta-rod test

2-1 Locomotion time /s

Number	CON	CPZ	20 mg/kg PB	100 mg/kg PB
1	130	48	111	160
2	111	36	93	170
3	120	69	94	136

4	114	60	115	113
5	160	91	101	110
6	95	86	100	114
7	128	50	98	139
8	128	58	131	137

2-2 Number of falls in 5 minutes

Number	CON	CPZ	20 mg/kg PB	100 mg/kg PB
1	1	9	8	3
2	5	12	3	6
3	2	7	4	8
4	3	8	7	5
5	5	11	7	3
6	6	13	10	6
7	4	8	5	4
8	2	5	8	3

3 Morris water maze

3-1 Escape lantencies during 4 days of training

DAY 1

Number	CON	CPZ	20 mg/kg PB	100 mg/kg PB
1	58.02	58.01	52.52	56.59
2	56.56	56.56	53.66	48.06
3	52.20	52.20	56.61	53.20
4	51.19	51.19	55.59	49.45
5	51.60	51.59	56.91	45.28
6	52.58	56.17	38.37	51.33
7	54.15	52.58	58.64	49.77
8	51.69	52.20	45.80	42.94

DAY 2

Number	CON	CPZ	20 mg/kg PB	100 mg/kg PB
1	23.25	49.25	38.50	36.00
2	39.00	48.00	29.80	43.01
3	41.25	50.00	38.60	37.02
4	37.75	50.00	37.50	41.00
5	28.25	60.00	35.80	36.03
6	48.75	46.30	45.25	38.50
7	28.25	60.00	40.50	30.00
8	25.68	41.50	48.20	50.00

DAY 3

Number	CON	CPZ	20 mg/kg PB	100 mg/kg PB

1	21.00	22.50	34.00	46.01
2	33.00	58.01	39.01	35.00
3	30.50	35.01	47.50	33.25
4	22.00	50.02	41.70	40.75
5	44.51	40.00	36.00	33.25
6	31.01	49.00	41.02	20.75
7	44.50	39.01	27.00	32.75
8	33.00	41.00	44.50	34.25

DAY 4

Number	CON	CPZ	20 mg/kg PB	100 mg/kg PB
1	9.00	38.00	33.00	21.75
2	17.33	34.33	30.50	21.25
3	11.67	45.33	46.25	20
4	10.33	34.58	36.25	31.67
5	28.33	42.00	31.75	30.00
6	24.00	38.00	34.50	19.00
7	43.67	33.55	28.02	30.67
8	18.01	34.33	36.41	25.00

DAY 5

Number	CON	CPZ	20 mg/kg PB	100 mg/kg PB
1	19.00	31.00	38.00	17.00
2	8.00	25.40	23.01	24.02
3	10.01	35.40	27.00	20.01
4	3.00	43.01	26.10	14.00
5	11.00	23.69	23.02	7.00
6	36.01	25.4	46.00	24.00
7	12.00	28.5	44.00	6.02
8	13.00	20.00	23.00	18.03

3-2 Distance in the target quadrant in probe test

Number	CON	CPZ	20 mg/kg PB	100 mg/kg PB
1	54.10	347.07	264.34	425.29
2	602.94	393.07	235.06	474.01
3	454.81	347.97	442.25	274.35
4	507.91	247.15	413.73	380.82
5	436.49	259.77	451.65	347.78
6	492.36	403.24	248.25	458.39
7	421.658	366.10	395.33	382.16
8	407.97	477.411	428.33	433.06

3-3 Time spent in the target quadrant in probe test

Number	CON	CPZ	20 mg/kg PB	100 mg/kg PB
1	25.52	15.42	15.25	20.58

2	27.50	15.17	16.99	20.05
3	40.55	18.94	17.21	25.19
4	20.31	18.80	25.62	23.07
5	33.53	17.08	19.14	25.27
6	25.65	21.24	19.18	27.24
7	23.15	21.22	12.36	18.22
8	24.12	19.20	10.36	19.66

3-4 Crossings into the former location of the platform

Number	CON	CPZ	20 mg/kg PB	100 mg/kg PB
1	2	0	1	2
2	5	1	0	2
3	3	2	4	
4	2	0	1	1
5	2	1	4	1
6	1	2	3	3
7	4	0	2	2
8	0	2	0	3

3-5 Swimming speed in probe test

Number	CON	CPZ	20 mg/kg PB	100 mg/kg PB
1	23.04	25.92	24.54	26.08
2	25.33	22.49	24.49	24.84
3	24.53	25.548	19.20	25.34
4	27.56	18.06	29.44	29.18
5	29.68	25.76	25.28	24.88
6	23.85	24.47	22.27	30.49
7	26.33	27.58	23.57	25.57
8	26.85	25.83	27.69	21.60
